# Research Progress and Clinical Application of All-Ceramic Micro-Veneer

**DOI:** 10.3390/ma16082957

**Published:** 2023-04-07

**Authors:** Zhenwei Ye, Jiapei Jiang, Linxin Yang, Tingxiang Xu, Yuanyou Lin, Feng Luo

**Affiliations:** 1 The State Key Laboratory Breeding Base of Basic Science of Stomatology (Hubei-MOST) & Key Laboratory of Oral Biomedicine Ministry of Education, School & Hospital of Stomatology, Wuhan University, Wuhan 430079, China; 2 Outpatient Department, Sichuan Electrical Power Hospital, Chengdu 610021, China; 3 State Key Laboratory of Oral Diseases, National Clinical Research Center for Oral Diseases, West China School of Stomatology, Sichuan University, Chengdu 610041, China

**Keywords:** minimally invasive, aesthetic, micro-veneer, treatment planning, tooth preparation

## Abstract

Anterior teeth problems affect the patient’s daily eating, communication, social activities, self-confidence, and mental health. The trend in dentistry is to address anterior tooth problems with minimally invasive and aesthetic treatments. With the development of adhesive materials and ceramics, micro-veneers have been proposed as an alternative treatment for enhancing the aesthetic appearance and avoiding unnecessary tooth reduction. A micro-veneer is a veneer that can be cemented to the surface without or with minimal tooth preparation. These benefits include no need for anesthesia, postoperative insensitivity, good adhesion to enamel, reversibility of treatment, and higher patient acceptance. However, the micro-veneer repair is suitable only for specific cases and must be strictly controlled regarding indication. Treatment planning is a crucial step to achieving functional and aesthetic rehabilitation, and following the clinical protocol is helpful for the longevity and success of micro-veneer restorations. However, more precise and predictable tooth preparation methods, such as minimally invasive microscopic tooth preparation and digitally guided veneer preparation, are recommended rather than the traditional free-hand method. Therefore, this paper clarifies micro-veneers and compares them with other restorations to gain a deeper and more comprehensive understanding. The authors also review indications, materials, cementation, and effect evaluation of micro-veneers to provide clinicians with valuable information. In conclusion, micro-veneers are minimally invasive treatments that provide good restoration results when used appropriately and are worthy of promotion for the aesthetic restoration of anterior teeth.

## 1. Introduction

In today’s world, patients are more conscious of aesthetic appearance due to increased living standards and awareness of oral health care. The presence of anterior teeth problems, including discoloration, loss, defect deformity, etc., can negatively impact a patient’s diet, communication, social activities, self-esteem, mental health, smile, and appearance [1]. Therefore, dentists and patients are increasingly focused on aesthetic dentistry, especially when it comes to aesthetic tooth restorations. At present, aesthetic dentistry aims to maintain and enhance the smile and correct differences in facial contour and jaw structure in a minimally invasive way [2].

The growing trend in dentistry is to perform minimally invasive treatments and aesthetically restore function [3]. There are many benefits to no or minimal preparation, including preservation of tooth structure without postoperative sensitivity, bonding to enamel, reducing flexural stresses, avoiding provisional, and improving patient satisfaction. Crowns, bridges, and veneers are commonly used all-ceramic aesthetic restorations for anterior teeth. In those aesthetic restorations, specific amounts of tooth tissue are removed and replaced with ceramic restorations. However, a micro-veneer is a veneer that can be cemented to the surface without or with minimal tooth preparation, dissimilar to traditional veneer [4]. With the development of adhesive materials and ceramics, micro-veneers have been proposed as an alternative treatment for enhancing the aesthetic appearance and avoiding unnecessary tooth reduction. Due to their tooth-colored appearance, they give patients a fully-adorning and natural-looking smile. Compared to resins, veneers are a more permanent option, last longer, and require no removal after installation [5]. A dental veneer can last for decades without needing to be replaced. Therefore, doctors and patients are increasingly interested in the minimally invasive aesthetic repair method of micro-veneers for the treatment of anterior teeth and for obtaining pleasing aesthetics and functionality. For example, Peng et al. described minimally invasive rehabilitation for severely worn dentition using computer-aided design and computer-aided manufacturing (CAD-CAM) veneers [6]. Furthermore, the advancement of enamel bonding techniques has led to an increase in the number of cases reporting micro-veneer aesthetic restorations without tooth preparation or with minimally invasive preparations. For instance, Butler et al. prepared 28 full-contour veneers for a patient with severe non-carious tooth surface loss using a minimally invasive tooth preparation [7]. A 13-month follow-up revealed no postoperative sensitivity, secondary caries, loss of tooth vitality, or chipping or fractures of the veneers.

Micro-veneers have become a hot topic in clinical research because of their minimal invasion and superior aesthetics. However, there is still no systematic elaboration of micro-veneers. Therefore, this paper clarifies micro-veneers and compares them with other restorations to gain a deeper and more comprehensive understanding. The authors also review indications, materials, cementation, and effect evaluation of micro-veneers to provide clinicians with valuable information.

## 2. The indications of Micro-Veneer

### 2.1. The Definition of Micro-Veneer

Minimally invasive dentistry (MID) was introduced in 1987 by Simonsen, and its citation frequency has steadily increased over time [8]. MID is a dental technique adapted to preserve the soundness and integrity of teeth while causing as minor damage as possible to the remaining teeth [9]. The trend may be mainly attributed to new dental bonding materials and more conservative techniques for healthy tooth tissue, but it is also due primarily to clinicians and patients with new mindsets. Korkut et al. demonstrate a minimally invasive, long-lasting, functional, and natural-looking smile makeover using direct composite veneers in a single appointment [10].

Dental veneers are thin, tooth-colored shells made from porcelain, ceramics, or acrylic and applied to the surface of a tooth to treat teeth with mismatched colors or shape alterations. Veneers are placed by removing a certain amount of tooth tissue and filling the space with ceramic. Although veneers are minimally invasive aesthetic restorations and the amount of tooth preparation for veneers is much smaller than that for full crowns, they still require some preparation of the teeth [11]. The ceramic veneers with thicknesses between 0.3 mm and 0.7 mm, combined with enamel bonding, are considered reliable. With the development of enamel bonding and ceramic materials, more and more dentists prefer conservative, minimally invasive methods for treating deformed anterior teeth with minor defects or excessive interdental spaces. They also lead to the appearance of micro-veneers.

Here, we refer to partial veneers, ultrathin veneers, “minimal preparation,” or even “no preparation,” as micro-veneers to distinguish them from full, regular, traditional veneers, which can be cemented onto tooth surfaces after no or minimal tooth preparation [12,13]. The indications for micro-veneers are similar to those for porcelain veneers. However, they are slightly more severe, and their range of indications is somewhat narrower. Generally, micro-veneers are indicated in anterior regions by slight coronal reshaping, small class III, IV, and V defects, diastemata, enamel micro-fractures, chippings, and slight discolorations [14]. Micro-veneers may be used conservatively to improve the teeth’ form, color, and function with a minimally invasive or no-preparation technique. However, due to their small thickness and high permeability, micro-veneers have limited effectiveness in improving tooth discoloration. This paper reviews the indications, materials, cementation, and effect evaluation of micro-veneers to help clinicians make more informed and effective clinical decisions.

### 2.2. The Indications of Micro-Veneers

When given a choice, patients are willing to opt for minimal tooth structure removal while achieving aesthetic and functional treatment goals at the same time. The ceramic micro-veneer can restore minimal morphological abnormalities in the anterior teeth while preserving the enamel to the most significant degree possible. For example, Ojeda et al. reported using partially laminated veneers to restore a maxillary right central incisor to match the more damaged adjacent tooth [15]. However, the micro-veneer repair method is only suitable for specific cases. Thus, the essential point of clinical practice is case selection. In addition, clinical details such as conservative tooth preparation, appropriate ceramic materials, appropriate cement, and the polishing of restorations should be addressed [16,17].

We reviewed the different aspects of micro-veneers with resins, full crowns, and veneers to gain a more comprehensive and detailed understanding of the process. As shown in Table 1, the indications of composite resin, micro-veneers, and veneers are the same. Perhaps this can be attributed to the similarity in restoration purpose and materials for composite resin, micro-veneer, and veneer [18]. There is no doubt that the resin pattern coincides with the micro-veneer pattern, both of which are minimally invasive to preserve as much of the natural tooth structure as possible. In addition, they offer some protection to patients because they are reversible and reparable.

The micro-veneers follow a minimally invasive principle, do not require systematic tooth preparation, and sometimes do not fully cover the labial surface of the abutments. Clinically, the micro-veneer thickness is sometimes too thin to conceal discolorations completely because of case selection and material limitations. So, micro-veneers are not suitable for severely discolored abutments. In general, micro-veneers are suitable for people with normal or mildly to moderately abnormal tooth colors [19]. Despite that, it only improves the color of abutment teeth and cannot alter or cover the color of many abutments themselves (severely discolored teeth). Additionally, micro-veneers are not recommended as the first-choice treatment for patients with bruxism or hyperocclusal forces due to the small amount of tooth preparation required for micro-veneers [20]. Moreover, the occlusal system should be carefully evaluated and managed when selecting micro-veneered restorations [21]. Herein, we summarized the indications and contraindications of the micro-veneer (Table 2).

Additionally, orthodontic therapies are helpful in repositioning teeth and reducing axial inclination to minimize the degree of surgical intervention required for veneer preparation in some cases [22,23]. For example, typical diastemas should be treated conservatively to protect the tooth structure. However, asymmetrical diastemas require a more complex treatment plan and may require a reallocation of space. Cunha et al. described a case in which ceramic micro-veneers served as an esthetic solution for asymmetric diastemas with minimal tooth preparation required [24]. The spaces of asymmetric diastemas were rearranged by moving peg-shaped maxillary lateral incisors with elastic separators. They then managed the horizontal distance during the rehabilitation of the smile with ceramic micro-veneers.

Nevertheless, no- or minimal-preparation veneers should only be considered after thoroughly evaluating their functionality and aesthetics. Swati Pralhad et al. demonstrate the importance of treatment planning to achieve good outcomes in diastema closure using minimally invasive indirect veneers [25]. Researchers also evaluated the clinical outcomes of minimally invasive ceramic restorations performed in 2021 by 16 professional dentists with different levels of experience [26]. The fractographic analysis demonstrated the external surface of restorations as the critical area and suggested that failures might occur due to noncompliance with the clinical protocol. Herein, we summarized the characteristics of the four restoration methods: resin, the full crown, veneer, and micro-veneer, according to their clinical application (Table 3) [9,10,11,12,13,14,15,16,18,19,20]. The characteristics included the amount of tooth preparation, remaining tooth tissue, resistance form, side reaction, aesthetic performance, patient acceptance, the incidence of adverse reactions, technical requirements, edge line, cementation requirements, and shedding rate of those restoration methods were different. For example, micro-veneers require only a tiny amount of tooth preparation, veneers and resins require less and full crowns require much more. Their characteristics in the clinical application are different. Further, the remaining tooth tissue for the micro-veneer is integral, the aesthetic performance is good, and the patient’s acceptance is high.

### 2.3. The Treatment Plan of Micro-Veneers

Treatment planning is a crucial step to achieving functional and aesthetic rehabilitation. A clinical guideline-based approach to diagnosing, planning, and executing ceramic micro-veneers is helpful for excellent aesthetic results and long-term success [27]. For example, planning each clinical case using a photographic protocol provides better predictability of the outcome [28]. Apart from photos and videos, the wax-up/model binomial is another essential tool that ensures the new smile’s symmetry and proportion [29]. Rocha et al. investigated color satisfaction, tooth shape satisfaction, marginal fit, second caries, and the sensitivity of minimally invasive ceramic restorations performed by dentists with different experience levels [26]. The clinical outcomes demonstrated that following the clinical protocol is essential for the longevity and success of micro-veneer restorations. D’Arcangelo et al. presented a new protocol to optimize micro-veneer restorations, which can improve marginal accuracy and the resulting aesthetic stability [30]. For specific esthetic concerns, such as multiple diastemas, tooth shape, and color problems, they also presented a step-by-step clinical protocol for diagnosis, treatment planning, and execution of minimally invasive micro-veneers [31]. In this case, micro-veneers were used to improve the aesthetics of the patient’s maxillary teeth, mainly regarding the multiple diastemas and color and shape of the teeth. After 2.5 years of follow-up observation, all veneers were still in exceptional condition, and periodontal tissue was healthy. The present clinical case demonstrates that micro-veneer repair using a step-by-step clinical protocol has good clinical results in restoring the patient’s aesthetics, function, and self-esteem.

In addition, the preparation design significantly affects ceramic micro-veneers’ fracture resistance and longevity. Blunck et al. investigate the influence of five different preparation designs on the margin quality and fracture resistance of ceramic laminate veneers after thermomechanical loading in vitro [32]. This study randomly assigned central incisors to various preparation designs, including non-prep, minimally invasive, semi-invasive, and invasive. A light microscope was used to examine the veneers for cracks, chips, partial fractures, and catastrophic fractures. The results indicated that ceramic laminate veneers are incredibly durable, and preparation designs are the main risk factors for fracture. Furthermore, Angerame et al. reported two preliminary designs for occlusal veneers fabricated from lithium disilicate glass-ceramic that resulted in similarly satisfactory results [33]. This study demonstrated that a new minimally invasive technique for occlusal veneer preparation with marginal chamfer showed good fracture resistance and marginal adaptation comparable to the conventional method. In addition, they emphasize that more enamel can be preserved when using this method for the preparation of micro-veneers. It is well known that micro-veneers bind better with more enamel remaining on the surface because the interface between porcelain and enamel is more accessible to bonding than the interface between porcelain and dentin. Previous studies have also demonstrated that abutments with unprepared teeth that retain the most significant amount of enamel achieve the best bonding and the highest shear forces [34].

**Figure 1:**
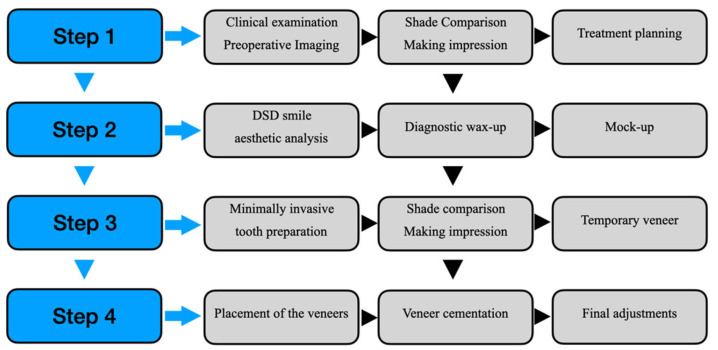
The clinical visit and operation flow chart for micro-veneer. Step 1 mainly collects patient data and makes a preliminary plan. Step 2 is to create an aesthetic design based on the information. Step 3 is to make the necessary preparations for the affected tooth and collect the data about the affected tooth again. The final step is bonding the prosthesis and finishing.

Presently, more and more clinical cases require non-prep or minimal tooth preparation to perform successful bonding, color transition, and contouring for veneers [17,35]. Figure 1 is a schematic diagram of the micro-veneer clinical visit and operation process protocol [10,12,27,28,30,31]. Step 1 mainly involves collecting patient data and making a preliminary plan. Step 2 is to create an aesthetic design based on the information. Step 3 is to make the necessary preparations for the affected tooth and collect the data about the affected tooth again. The final step is bonding the prosthesis and finishing. The second and third visits can be combined if the patient communicates, agrees, and has sufficient time. Impression materials can use alginate, silicone rubber, polyether rubber, a 3-shape oral 3D scanner, and an oral cavity model scanner to take molds.

### 2.4. Preparation Methods for Micro-Veneer

Incorrect clinical treatment design and tooth preparation can result in adverse effects, including decreased adhesive strength of micro-veneers, increased dentin sensitivity, microleakage, and poor long-term outcomes [31,36]. Therefore, a more precise and predictable method of tooth preparation rather than the traditional free-hand method should be utilized to prepare micro veneers due to their small restoration space and tooth preparation volume.

Specialists recommend minimally invasive microscopic tooth preparation for cosmetic restorations with micro-veneers. With microscopic tooth preparation, dentists can magnify the teeth up to 20 times more and have better illumination power than the conventional free-hand method [37]. This technique helps obtain a precisely designed preparation amount to preserve the maximum amount of preparation, obtain a reasonable shape of the abutment tooth, and create the necessary space for realizing the goal of the restorative procedure. Eichenberger et al. evaluated the effect of magnification on tooth preparation under clinically stimulated conditions using standardized procedures [38]. There were significant differences between preparations made using an optically sophisticated operating microscope, Galilean loupes with coaxial illumination, and preparations made with the naked eye. Dental magnification devices allow dentists to precisely identify and treat delicate tooth structures in a simulated clinical setting.

Furthermore, the digitally guided veneer preparation system is another recommended method for the controlled and predictable preparation of micro-veneer [39]. For instance, Marques S et al. proposed incorporating computer-aided design (CAD) and computer-aided manufacture (CAM) technology into the preparation of ceramic veneers [40]. By using a digitally guided veneer preparation system, a controlled, selective, and noninvasive veneer preparation can be performed based on the approved trial restoration technique. Nonetheless, it is limited to the minimum thickness necessary for the final ceramic restoration. Silva et al. describe a minimally invasive and efficient method to achieve predictable, accurate results using digital CAD-CAM technology [41]. A new digital technology, the first fit system, was introduced in this study to allow precise measurement and control of the reduction of tooth structure during the preparation of ceramic veneers. In the first fit system, veneers are prepared in a one- or two-step process using 3D-printed guides and a special handpiece. Peng et al. used digital technologies to facilitate minimally invasive rehabilitation using CAD-CAM ceramic veneers for severely worn dentition [6]. In this case, the lingual veneers without tooth preparation and the occlusal veneers with minimal tooth preparation were designed from the digital scan of the interim restorations. The digital technique provided a valid and reliable method for making and cementing multiple CAD-CAM veneers to restore worn teeth minimally invasively. In conclusion, a digitally guided veneer preparation workflow may allow for more accurate tooth structure preservation since virtual preparation is performed with a CAD program.

### 2.5. Repair Space Required for Micro-Veneer

During the selection process, it is crucial to identify whether the abutment has “available space for external restoration outside of the dental tissue” [42]. The restoration space is an important indicator of whether the abutment needs to be prepared. “External restoration space” refers to the space between the abutment to be restored and the final design restoration position. The minimally invasive space is also built on “external restoration areas.” In this case, “targeted restorative space” can be used to assess “external restoration space” [37]. Tooth preparation provides the restoration with a suitable space outside the dental tissue to be positioned close to the abutment.

In specialized settings, minimally invasive spaces are preferable to minimally invasive methods. If enough external restoration space is available and sufficient, micro-veneer restorations can be performed with less preparation or even without it. In contrast, tooth preparation is required if it is insufficient or not enough “external restoration space” for micro-veneer restoration, tooth preparation is required. Minor tooth defects can be repaired by removing only sharp angles, protrusions, and undercuts that interfere with the restoration. Then the minimally invasive restoration can be completed. Insufficient attention to sharp angles will result in cracking, which may expand as the bonding process proceeds [43]. Furthermore, tooth preparation may not be necessary if the inherent undercut does not interfere with the restoration seating.

For micro-veneer materials, the thickness is generally 0.3–0.5 mm, indicating that the “external restoration space” has to reserve at least 0.3–0.5 mm for material processing as a minimum [44]. Gierthmuehlen et al. evaluated the effect of ceramic thickness on fatigue survival and failure loads of minimally invasive veneers. Results showed that the monolithic lithium disilicate veneer restoration with a thickness of 0.5/0.4 mm had an overall fatigue survival rate of 98.1% and exceeded physiological mastication forces [45]. Although 0.3–0.5 mm would theoretically be sufficient to meet specification requirements, restorations are usually not as thin as theoretically required. For instance, Unkovskiy et al. used lithography-based ceramic manufacturing (LCM) technology to print 0.1 mm-thick non-prep ceramic veneers successfully and demonstrated a good fit for the model in vitro [45]. There does not appear to be an objective system for measuring the “external restoration space” in clinical work. Usually, they are based on clinicians’ subjective evaluations and the auxiliary design of visual expressions, such as digital smile designs, wax-ups, and mock-ups [46].

### 2.6. Materials Used for Micro-Veneer

The trend toward minimally invasive dentistry has resulted in the introduction of micro-veneers for cases requiring little dental work in the front teeth, reshaping the teeth, such as closing diastemas or treating microcracks, minor discolorations, and enamel surface defects [15]. Currently, two types of veneer are commonly used for micro-veneer: composite resin and ceramic [47,48].

Composite veneers are directly applied to minimally prepared tooth surfaces using composite resin. It can be accomplished in a single dental appointment, and the dentist can easily modify the restoration’s shade, size, and shape. Lippert et al. demonstrate the effectiveness of complete oral rehabilitation with composite resins using indirect application and direct techniques for severely worn teeth. In their opinion, either directly or indirectly, composite resins enable the recovery of function and aesthetics without damaging the teeth and reduce financial investment [49]. However, direct composite resin veneers have some disadvantages, including the likelihood of replacement due to wear, loss of anatomical shape, and long-term color stability. The surface gloss of the resin is susceptible to alteration and potential discoloration. Further, the fracture is another factor contributing to the failure of composite repairs. A randomized, split-mouth clinical study evaluated direct composite resin veneers over three years and reported an overall survival rate of 87.5% [50].

Ceramic materials have advantages over composite resin, such as their outstanding optical properties, biocompatibility, low thermal conductivity, color stability, and excellent mechanical properties [51]. Ceramic veneers’ longevity and success rate are better than composite resins. Aslan et al. evaluated the long-term clinical performance and survival rate of pressable lithium-disilicate glass-ceramic veneers using a retrospective case series study [52]. Among the 413 restorations, the incidence of fracture and debonding was only 6 (1.45%) and 9 (2.18%) in this study. In addition, the five-year survival rate of 413 veneers was 98%, the 10-year survival rate was 95%, the 15-year survival rate was 91%, and the 20-year survival rate was 87%, indicating that the clinical failure rate of ceramic veneers is very low. In addition, the ceramic undergoes less wear when compared to composite resin, and its color stability can endure up to ten years of clinical use [50]. Araujo et al. compared ceramics to composite resin for restoring teeth with anterior veneers utilizing a minimally invasive method and discussed the selection of materials and techniques for restoring anterior veneers [53]. The results suggested that ceramic veneers on anterior maxillary teeth performed significantly better than composite indirect laminate veneers after a decade in terms of survival rate and restoration quality.

Furthermore, an in vitro study also supported that laminate micro-veneers have fracture strength values comparable to direct composite restorations or conventional ceramic laminate veneers [23]. Gresnigt et al., 2020, investigated the fracture strengths and failure modes of laminate veneers, partial laminate veneers, and composite restorations after aging in vitro [54]. This study found that partially laminated veneers, even those that contained cracks after thermal cycling, performed similarly to control and conventionally laminated veneers in terms of fracture strength. More importantly, the success rate of micro-veneers is comparable to that of traditional veneers. Smielak et al. compared the survival rates of 186 conventional and no-prep/minimally invasive porcelain veneers in 35 patients [55]. After nine years of observation, the survival rate of no-prep/minimally invasive veneers was higher than that of conventional veneers. Therefore, micro-veneers can provide excellent results in specific clinical settings and should be seriously considered in the future. Dental restoration materials have gradually shifted from composite resins and alloys to ceramic materials such as silicate, polycrystalline, and resin-based ceramic.

In clinical practice, micro-veneers are rarely designed with a thickness of more than 1.2 mm. Thus, the materials used for micro-veneer are high-performance ceramic materials due to their mechanical and aesthetic properties that meet clinical needs. As reported, lithium disilicate-reinforced glass ceramic, feldspathic porcelain, and leucite-reinforced feldspathic glass ceramic are the common materials used for micro-veneer and veneer restoration. All-ceramic veneers are primarily made of hot die-cast ceramics [56]. For hot die-casting ceramics, third-generation ceramic materials such as IPS-E. Max or IPS-EMPRESS II, made from lithium disilicate glass matrix, are commonly used [57]. In addition to their high compressive strength, third-generation ceramics have good flexibility, reaching 360–400 Mpa, preventing mechanical problems such as chipping and falling off. It also has corrosion resistance and long-term temperature stability, reducing the risk of long-term wear-induced discoloration. The excellent biocompatibility of the material minimizes the impact on neighboring teeth and reduces sensitivity, gingival, and pulpal problems during and after the restoration process. Moreover, it has poor thermal conductivity, which means it is less resistant to cold and heat and offers better protection to the abutment’s dentin, pulp, and periodontal tissues.

## 3. The Cementation of Micro-Veneer

### 3.1. The Pretreatment of Micro-Veneer

Cementation materials and bonding techniques have steadily expanded ceramic’s indication range, making micro-veneers a highly viable alternative to more invasive, classic restorative treatments [58]. Micro-veneers’ longevity depends primarily on the strength and durability of the adhesive placed between the tooth surface, resin cement, and porcelain surface. The adhesive failure at the ceramic-cement interface will lead to the debonding of the micro-veneers.

The enamel layer is the primary and ideal cementing matrix [31,55]. Dawood et al. reported that the effect of different dental bonding substrates and bonding methods on marginal quality was evaluated in mandibular molars restored with monolithic zirconia occlusal veneers after thermal aging [59]. They recognized that after thermal aging, the type of bonding surface for zirconia occlusal veneers (enamel, dentin, dentin with composite filling, or dentin with intra-coronal cavity) could affect the marginal quality. The success of cementing porcelain veneers is determined by how well three components adhere to one another: cement, restoration, and dental tissue. It is necessary to conventionally pretreat the bonding surface before cementing [60]. Grinding, sandblasting, acid etching, laser etching, silicon coating, and silanization are common pretreatments for porcelain veneers before cementation.

The currently recognized acid etch-rinse system is the “gold standard” for enamel bonding [61]. Regarding surface treatment for porcelain veneers, hydrofluoric acid etching and silane coupling agents are the most effective [62]. Chemically etching ceramic surfaces with hydrofluoric acid can enhance surface roughness and luting agent reactivity. A silane coupling agent can enhance chemical bonding strength as well. For example, Kaushik et al. described two cases involving fractured teeth, sensitivity, discoloration, and malalignment of teeth treated with lithium disilicate ceramic veneers to achieve good aesthetic results with minimal invasiveness [63]. In these cases, an etching solution containing 9.5% hydrofluoric acid was applied to veneers, followed by a coupling agent containing silanes. For the cementation of veneers to the tooth structure, 37% phosphoric acid was used to etch the teeth, and dual-cure resin cement was used to etch the teeth. Further, Xiao et al. presented a clinical report with a 3-year follow-up using ceramic veneers to fulfill patient demand for aesthetic improvement without removing and replacing old restorations [64]. In this case, feldspathic ceramic veneers were hydrofluoric acid etched and silane coated to achieve micromechanical interlocking and chemical bonds to ensure successful ceramic-resin bonding.

What needs more attention is that the acid etching-rinsing system must thoroughly wash the acid etching agent in clinical use. The operation is cumbersome, which makes clinicians prefer to use a general-purpose adhesive. Fortunately, it is convenient to operate and can reduce technical sensitivity. In addition, it is recommended to use a magnifying glass or microscope for cementing [37]. Studies have shown that beginners operate better with a magnifying glass. There are situations in which some dentin is exposed due to morphological defects. The dentin and enamel adhesive systems can be combined when the adhesive system is used. Furthermore, compared with self-etch, the etch-and-rinse bonding method demonstrated the superior marginal quality of zirconia occlusal veneer restoration after thermal aging [59].

In addition, to ensure the long-term success of micro-veneers, careful selection of the cementation system and appropriate restorative procedures are essential. Resin cement is the commonly used material for the cementation of indirect restoration. Resin cement can be divided into self-curing, light-curing, and dual-curing cement [65]. Among them, light-cured and dual-cured cement is recommended for micro-veneer adhesive luting. Considering the wide variety of resin cement available, selecting suitable adhesives for long-term restoration retention is crucial [66]. Therefore, the effectiveness of shear bond strength estimation needs to be explored according to cement type (light/dual-cure) and restoration surface treatment. For example, the shear bond strengths of different luting cement bonding to pre-treated lithium disilicates were examined by Alkhurays et al., 2019 [67]. As reported, the shear bond strength of the micro-etch group was significantly higher across all cement tested than that of the acid-etch group (*p* < 0.05), suggesting the surface treatment impacts shear bond strength primarily regardless of the resin used. Thus, surface treatment influences the bond strength irrespective of the type of resin cement (light/dual-cure) used for indirect restorations’ cementation.

### 3.2. Thickness of the Adhesive

The thickness of the adhesive plays an essential role in the bone’s strength and shear strength. Generally, the adhesive thickness should be moderate, not too thin or too thick. Sampaio et al. evaluated the cement film thickness of veneers through microcomputed tomography [68]. According to Liu et al., cement thickness influences micromechanical and micromechanical responses in incisal-overlapped incisors adjacent to a ceramic veneer [69]. They use finite element analysis to investigate the effects of cement type and thickness on stress distribution inside ceramic crowns. The result indicated that higher elastic modulus cement causes lower tensile stresses in the veneer and core layers and that shear-strength cement is critical to ceramic restorations’ intactness. According to them, higher adhesive stress develops with increasing cement thickness. For this reason, cement thicknesses less than 0.5 mm may reduce the failure rate of adhesive bonding. However, it was reported that 0.1 mm to 0.2 mm of resin adhesive thickness is appropriate for clinical use [70]. Silami et al. (2015) assessed the color stability of resin cement when bonded to ceramic laminate veneers with different thicknesses under accelerated aging conditions [71].

Furthermore, microscopy and X-ray apparatus have been used to study the effects of adhesive penetration on adhesive bond performance for several decades [72]. In 2017, Tribst et al. evaluated stress distribution in an occlusal veneer for different materials, restoration thickness, and cement layer thickness [73]. The results showed that the cement layer thickness did not affect stress distribution in the restoration (*p* ≥ 0.10). Restorations with thicker restorations will result in higher tensile stress concentrations, but the cement layer thickness did not influence stress distribution in the restoration (*p* ≥ 0.10). However, in clinical practice, there is no precise objective index to use to determine the thickness of the adhesive and to ensure the adhesive’s uniform thickness on the micro-veneer’s adhesive surface.

### 3.3. The Color Effect of Micro-Veneer

Due to the porcelain material’s nature, the veneer’s required thickness is 0.2–0.3 mm for each color level increase. In some studies, researchers have found that a thickness of at least 1.5 mm is necessary to cover the abutment’s color altogether [74]. For instance, the thickness of the IPS Empress all-ceramic material must reach 1.6 mm to completely cover the base layer’s color [75]. When the thickness of the veneer is less than 1 mm, the expected change in the color of the abutment and the adhesive can be observed with the naked eye, and the apparent final color change can be seen [76]. Therefore, when using thinner porcelain veneers for restoration, the appropriate adhesive can be selected by the color of the abutment. For example, sometimes the enamel layer of the base tooth is insufficient, the dentin layer is exposed, and the color is dark. The optical transparency of all-ceramic veneers is good, which can easily lead to a darker color of the repair effect after cementing. For the above situation, color test pastes can be used to simulate the color after cementing, the veneer can be stained, or the ratio of different color bonding agents can be adjusted.

The aesthetic outcomes can be improved because of the high level of biomimetics in adhesive materials. For example, Ozisci et al. studied the effect of translucent adhesive resin cement on the final color of ceramic laminate veneer restorations before and after polymerization [77]. The in vivo study results suggest that the effect of light polymerization on the translucent resin cement is crucial to determining the final color of the restoration during shade selection and fabrication. Further, Batista et al. stated that the color change during ceramic veneer treatment was correlated with the thickness of the veneer and the tooth treated [78]. Khosravani et al. evaluated the effect of ceramic thickness and resin cement shade on the final color of different layers of ultra-translucent multilayered (UTML) zirconia veneers. They addressed the fact that the lower ceramic thickness showed a more remarkable color change. In addition, resin cement shades and layers of zirconia affected the color differently [79].

The final restoration color effect of porcelain veneer is affected by the interaction of the color of the abutment, the veneer, and the cementing material. We summarize the color-influencing factors that affect the effect of micro-veneer restorations (Table 4). (1) Abutment factors: location of discoloration, lightness of discoloration, hue of discoloration, saturation of discoloration; (2) veneer factors: type of material, transparency of veneer, thickness of veneer, craftsmanship; (3) adhesive factors: type, color, thickness, transparency. It shows that there are many minor points under the three influencing factors. In order to guarantee the color after repair, much attention must be paid during the treatment. The core influencing factors are veneer thickness, material transparency, and abutment color. The factors of adhesion can play a critical role in the adjustment.

## 4. Clinical Aspects of Micro-Veneer

Although micro-veneers require the expertise of doctors, a physician’s professional experience does not determine patient satisfaction or clinical success [26]. The aesthetic restoration effect of the micro-veneer is determined by the patient’s subjective aesthetic and clinical objective factors. The abutment’s condition directly affects the shape, thickness, and color of the micro-veneer restoration, and the cementing system ensures its long-term clinical outcome [80]. The evaluation of the veneer repair effect is divided into two parts: subjective and objective. The objective part is the evaluation index of clinical efficacy, and the subjective factor is the degree of patient satisfaction (aesthetic restoration of anterior teeth needs to be based on the patient’s aesthetics).

### 4.1. Effect Indicators

There are two mainstream clinical efficacy evaluation index systems: one is to refer to the American CDA standard system (including edge fit, edge coloring, restoration color, anatomical shape, secondary caries around restorations, and restorations that wear, fracture, and fall off); the second is the USPHS evaluation standard, that is, the modified US Public Health Service evaluation standard to evaluate the restoration effect (including integrity, translucency, color, edge coloring, and edge adhesion) [81]. These two mainstream evaluation index systems are similar. No matter which evaluation index system is used, many experimental studies show that the veneer edge of the restoration method without dental preparation is closely matched, the color transition is natural, and the color distribution is even [55]. Furthermore, Ojeda et al. used the modified USPHS criteria to evaluate the repair effect of the micro-veneer [18]. In addition, we summarized the general success or survival rate of the micro-veneers (Table 5). As reported, the results demonstrated that the success rate and survival rate of micro-veneer repair are relatively high (>90%) [12,82]. According to the objective clinical indicators, the micro-veneer is in line with the standard and is clinically recognized. 

### 4.2. Satisfaction

Most criteria for patient satisfaction are subjective evaluations or visual analog scales. The patients are delighted with the shape and color after restoration (>90%) [83]. Only a small number of patients are completely satisfied with the effect, which may be due to the poor transition of the restoration edge line [18]. Studies also show that rapid loss of composite resin cement from the edges of porcelain veneers appears to be the leading cause of failure, eventually leading to unsupported porcelain veneers breaking [84]. Dentists can perform detailed grinding and polishing by molding transparent ceramic and translucent resin materials on the edge of the prosthesis. Placing the prosthesis along the natural buccal convexity line can avoid excessive contour and edge differences. Furthermore, changing the protrusion contour ensures a natural transition between the restoration and abutment edges [82].

The edge line of the micro-veneer is mainly in the self-cleaning area. Traditional all-ceramic veneers need to grind more tooth tissue to form a long edge line and a tooth shape conducive to cementing. Due to the long edge line, the contiguous area is difficult to clean clearly, and the edge transition is not good. There is a possibility of secondary caries, which eventually leads to failure. Several situations are considered absolute failures, including secondary caries, fractures, debonding of indirect restorations, severe periodontal breakdown, and pulpal necrosis [13]. Following conventional tooth preparation, micro-veneers are more successful at restoring teeth than traditional porcelain veneers due to the integrity of the enamel, good adhesion, and reliable cementing system [55]. As Blunck et al. reported, even after three million cycles with up to 100 N, micro-veneers (non-prep or minimally invasive veneers) showed high survival rates [32]. The fracture risk increases with thin veneers and preparations with medium-to-high dentin portions compared to thicker veneers with preparations in enamel or partially in dentin (*p* ≤ 0.05). 

### 4.3. Limitation of Micro-Veneer

Micro-veneer restoration requires completing multiple technical steps such as practical aesthetic design, precise model making, and wax-up making. The procedure of the micro-veneer is operator-sensitive. All have high requirements for clinicians and dental technicians. Laboratory and clinic dental veneers are difficult to obtain in a relatively natural and harmonic shape and are prone to fracture. In addition, micro-veneers should be carefully recommended to patients who are very motivated to keep their teeth clean because such procedures require additional skills for delivering, finishing, and polishing porcelain within the mouth. The gingival reaction is one of the typical adverse reactions to restoration [85]. Its appearance and incidence rate are shallow and may not even appear within 12 months of follow-up in some studies. The gingival reaction may be closely related to non-preparation and the integrity of tooth enamel. The primary reported micro-veneer complications included secondary caries, pigmentation of the margins, bleeding on probing, fractures, and loss of retention. For instance, the bonding procedure may lead to micro-veneer fractures and affect the micro-veneer’s survival rate. Another critical point is determining the location of the best edge: the edge is at the point of maximum convexity of the tooth to ensure a smooth transition [41].

The disadvantages of micro-veneers need to be noted, including limited restoration space and difficulty in determining the margin line of the restoration. Other clinical details should be considered, such as conservative tooth preparation, appropriate ceramic material selection, appropriate cementing materials, and appropriate polishing. Micro-veneers are the preferred treatment for mild and moderately discolored teeth, but not severely discolored ones. These shortcomings are mainly reflected in the limitations of the indication range and shading effect. Moreover, the occlusal system should be assessed and managed when selecting a micro-veneer restoration. Therefore, adhering to an analytical treatment concept is essential to implementing the no-prep veneer technique successfully.

## 5. Conclusions

The growing trend in dentistry is to perform minimally invasive treatments and aesthetically restore function. Micro-veneer is a veneer that can be cemented to the surface with or without minimal tooth preparation, dissimilar to traditional veneer. The characteristics of micro-veneers can be summarized as follows:

- No need for anesthesia.
- No or minimal preparation is needed.
- The cementation protocol is critical.
- It is a reversible treatment.
- It has a high level of acceptance by the patient.
- It needs a follow-up protocol.
- Can be repaired in very specific cases.
- Can correct minor defects in shape.
- Can correct slight color changes.
- Can correct mild functional defects.
- Requires a high level of expertise on the part of the practitioner.

In conclusion, micro-veneers are minimally invasive treatments that provide good restoration results when used appropriately. It deserves promotion because it restores anterior teeth in an aesthetic and minimally invasive manner.

## Figures and Tables

**Table 1:** The difference of indications for resin, the full crown, veneer, and micro-veneer.

Indications	Resin	Full Crown	Veneer	Micro-Veneer	References
Tooth defect	mild to moderate	severe	mild to moderate	mild to moderate	[6,7,11]
Abnormal shape	mild	severe	mild to moderate	mild to moderate	[5,12]
Abnormal color	mild to moderate	severe	mild to moderate	mild to moderate	[10,18,19]

**Table 2:** The indications and contraindications of micro-veneer.

Indications	Contraindications	References
mildly to moderately abnormal color	severely abnormal color	[3]
mild to moderate tooth defect	severely tooth defect	[7]
mildly irregular tooth alignment abnormal morphology of the tooth	severely irregular tooth alignment severe lack of required space	[18]
widened space between teeth	Oral habits	[5,12]

**Table 3:** The characteristics of four restoration methods: are resin, full crown, veneer, and micro-veneer.

Characteristics	Micro-Veneer	Veneer	Full Crown	Resin
Amount of tooth preparation	minute quantity	Less	much	Less
Remaining tooth tissue	integral	Much	less	Integral
Resistance form	poor	Poor	good	Poor
Side reaction	less	Less	much	Less
Aesthetic performance	good	Good	good	Medium
Patient acceptance	high	High	low	High
Incidence of adverse reactions	low	Low	low	Low
Technical requirements	high	high	high	Low
Edge line	shorter	long	short	Long
Cementation requirements	high	high	low	High
Shedding rate	low	low	medium	High

**Table 4:** The color-influencing factors that affect the effect of micro-veneer restorations.

Abutment Factors	Veneer Factors	Adhesive Factors	References
Location of discoloration	Type of material	Type	[72,77,78]
Lightness of discoloration	Transparency of material	Color	[74,79]
Hue of discoloration	Thickness of veneer	Thickness	[58,68,69,73,75]
Saturation of discoloration	Making craftsmanship	Transparency	[76,79]

**Table 5:** The success or survival rate of micro-veneer.

Author	Success or Survival Rate (%)	Observation Period	Reference
Mihali S G, et al., 2022	91.77	84 months	[13]
Lz N, et al., 2022	95.9	60 months	[18]
Passos Rocha, E. et al., 2021	94.5	12 months	[26]
Gonzalez-martin O. et al., 2021	90.20	36 months	[35]
Aslan Y U. et al., 2019	98	60 months	[50]
Smielak B. et al., 2021	100.00	108 months	[55]
De Angelis F. et al., 2021	97.40	43.1 months	[82]

## Data Availability

Not applicable.
